# Polymerase-Endonuclease Amplification Reaction (PEAR) for Large-Scale Enzymatic Production of Antisense Oligonucleotides

**DOI:** 10.1371/journal.pone.0008430

**Published:** 2010-01-01

**Authors:** Xiaolong Wang, Deming Gou, Shuang-yong Xu

**Affiliations:** 1 Department of Biotechnology, Ocean University of China, Qingdao, Shandong, People's Republic of China; 2 Department of Pediatrics, University of Illinois at Chicago, Chicago, Illinois, United States of America; 3 New England Biolabs Inc., Ipswich, Massachusetts, United States of America; University of Helsinki, Finland

## Abstract

Antisense oligonucleotides targeting microRNAs or their mRNA targets prove to be powerful tools for molecular biology research and may eventually emerge as new therapeutic agents. Synthetic oligonucleotides are often contaminated with highly homologous failure sequences. Synthesis of a certain oligonucleotide is difficult to scale up because it requires expensive equipment, hazardous chemicals and a tedious purification process. Here we report a novel thermocyclic reaction, polymerase-endonuclease amplification reaction (PEAR), for the amplification of oligonucleotides. A target oligonucleotide and a tandem repeated antisense probe are subjected to repeated cycles of denaturing, annealing, elongation and cleaving, in which thermostable DNA polymerase elongation and strand slipping generate duplex tandem repeats, and thermostable endonuclease (PspGI) cleavage releases monomeric duplex oligonucleotides. Each round of PEAR achieves over 100-fold amplification. The product can be used in one more round of PEAR directly, and the process can be further repeated. In addition to avoiding dangerous materials and improved product purity, this reaction is easy to scale up and amenable to full automation. PEAR has the potential to be a useful tool for large-scale production of antisense oligonucleotide drugs.

## Introduction

MicroRNAs (miRNAs) are a family of short noncoding regulatory RNA molecules. The miRNA pathway serves as an important post-transcriptional regulation mechanism [1]. Synthetic antisense oligonucleotides targeting miRNAs or their mRNA targets are proving to be powerful tools for molecular biology research [2] and may eventually find application as new therapeutic agents [3], [4].

Large quantities (from multi-grams to kilograms) of a specific oligonucleotide have to be produced for commercial production of antisense oligonucleotide drugs. Therefore, the development of an economical and safe method for industrial production of short oligonucleotides has become necessary. Traditional method for *de novo* oligonucleotide synthesizing is the phosphodiester method [5]. Using automatic synthesizers, micrograms to kilograms of a specific oligonucleotide can be produced in a few hours. Unfortunately, synthetic oligonucleotides are often contaminated with a significant fraction of truncated failure sequences, makes the product purification process difficult. Moreover, the oligonucleotide synthesis process requires not only expensive equipments, but also costly and hazardous chemicals. For example, an organic solvent (dichloromethane or toluene) must be used to dissolve the deblocking reagent, which arises the problem of disposing chemical wastes.

In modern molecular biology laboratories, nucleic acids are routinely amplified by polymerase chain reaction (PCR). PCR has the limitation, however, of needing a pair of synthetic primers. This imposes not only a length constraint on the target DNA, but also a yield limit on the PCR product defined by primer concentrations. Therefore, PCR-based methods are generally not applicable for the amplification of short oligonucleotides that are only approximately 20 nucleotides (nt) in length. Enzymatic amplification of oligonucleotides has been reported by successive rounds of ligation, rolling circle replication (RCR) and cleaving [6] or nicking [7], but a laborious recircularization process is required for each round of RCR. Here we report a novel thermocyclic reaction, polymerase-endonuclease amplification reaction (PEAR), for amplification of oligonucleotides. In addition to avoid using hazardous chemicals and improving product purity, this reaction is easy to scale up and amenable to full automation, thus enabling large-scale and pollution-free antisense oligonucleotide production.

### The Principle of PEAR

A PEAR reaction contains a target oligonucleotide (*X*), an antisense probe, a thermostable DNA polymerase (such as Taq polymerase), a thermostable restriction endonuclease (such as PspGI), four dNTPs and an appropriate buffer solution. The antisense probe, denoted by *X′R′X′*, is designed to be a single-stranded oligonucleotide containing at least two tandem repeated complements of the target sequence (*X′*) that are separated from one another by an intervening complementary recognition site (*R′*) for PspGI.

**Figure 1:**
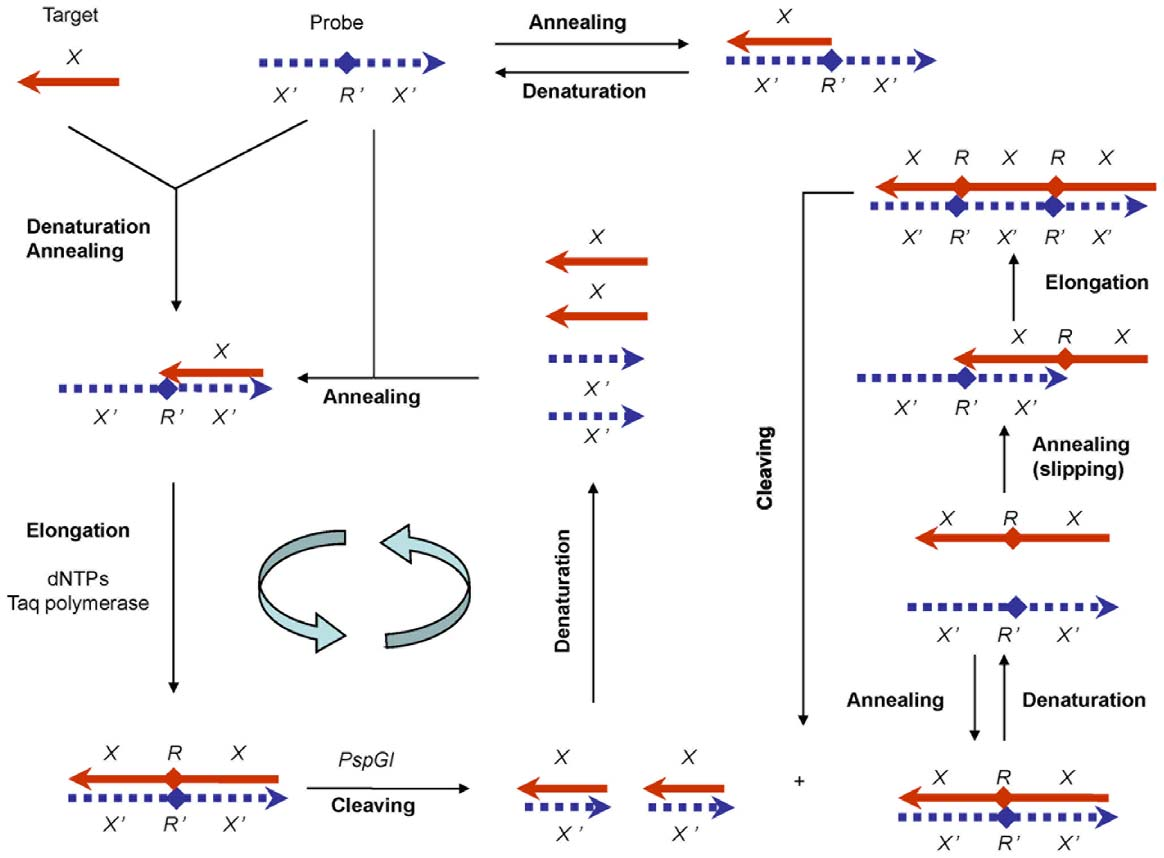
Schematic description of PEAR. Sense and antisense strands are represented by solid and dashed lines respectively, the 3′ ends are indicated by arrows and the restriction sites for PspGI are indicated by solid diamonds. When a target oligonucleotide (*X*) binds to a probe in the upstream, it is elongated by the Taq DNA polymerase, and a full-duplex oligonucleotide containing tandem repeats is produced. If the repeats are cleaved by PspGI, short duplex oligos (*X/X′*) are released; and when they are not cleaved, the number of tandem repeats increases by slipping and elongation.

As shown in Fig 1, PEAR consists of repetitive cycles of: (1) heat denaturation, (2) annealing, (3) elongation, and (4) cleaving. In the first cycle, a target oligonucleotide and an antisense probe were heat-denatured and annealed to form a partial duplex (*X/X′R′X′*). When a target oligonucleotide binds to a probe in the upstream (Fig 1, top right), there is no elongation, because it provides no primer/template structure for the Taq polymerase. However, as both target and probe are present in a large number of copies, according to the law of probability, nearly half of the target oligonucleotides bind to the probe in the downstream. In the presence of dNTPs, they are elongated by Taq DNA polymerase to form fully matched duplex tandem repeats (*XRX/X′R′X′*). Subsequently, PspGI cleavage of the recognition site releases monomeric oligonucleotides (*X/X′*). Thereafter, a next cycle of denaturation, annealing, elongation and cleaving is started again, resulting in exponential amplification of the duplex oligonucleotide.

In addition, the tandem repeated duplexes are not fully digested by PspGI, because the duration of cleavage is rather short. When the remaining tandem repeated duplexes are subjected to more cycles of denaturing, reannealing and elongation, the number of repeat units increases continuously through slipped strand pairing and DNA polymerase elongation (Fig 1, right). When PspGI cleavage monomerizes the elongated tandem repeats in a following cycle, many more duplex oligonucleotides are released. It is this *slipping-and-cleaving mechanism* that promotes not only the rate of amplification, but also the yield of product. Moreover, the PEAR products can be used in a next round of PEAR amplification directly without any treatment, and the process can be further repeated. The tandem repeated oligonucleotides are thus called *seeds*, because they can reproduce themselves.

## Results

### Implementation of PEAR

**Figure 2:**
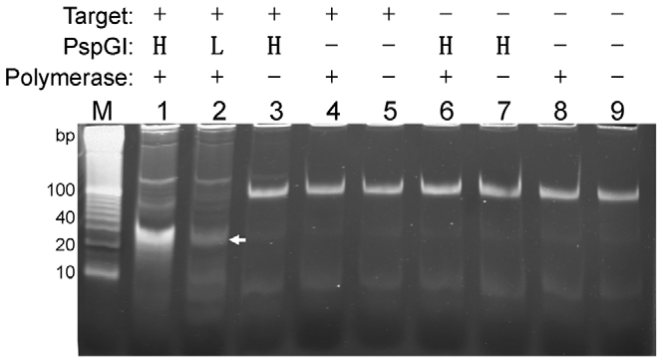
PEAR reactions with complete and incomplete components. Target (X) and probe (X′R′X′R′X′) concentrations were at 1nM and 100 nM respectively. For PspGI, H and L stand for high (0.4 U/µL) and low (0.1 U/µL) concentrations respectively. Lane M: Invitrogen Trackit™ 10 bp DNA ladder; Lane 1–2: complete PEAR reactions containing Taq DNA polymerase, PspGI, the target and the probe. The lower band (shown by an arrow) represents the 20-bp duplex monomers, and the upper bands represent tandem repeats; Lane 3–9: incomplete PEAR reactions lacking one or both enzymes or the target. No product band was observed. The bands represent probe self-dimerization formed by intermolecular interactions.

A synthetic oligonucleotide and an antisense probe derived from human microRNA miR-375 were used to validate the proposed reaction mechanism. PEAR reactions with complete and incomplete (lacking Taq DNA polymerase, PspGI or target) components were conducted under previously optimized reaction conditions with target concentration at 1 nM and probe concentration at 100 nM. As shown in Fig 2, a lower band represents the duplex product *X/X′* and several upper bands represent tandem repeats are observed in the complete PEAR reactions, but such bands are not observed if any of the four essential components, the two enzymes, the target and the probe, is omitted. We further tested another three pairs of targets and antisense probes, and the amplifications are all dependent on the presence of two enzymes, the target, and the probe (data not shown).

**Figure 3:**
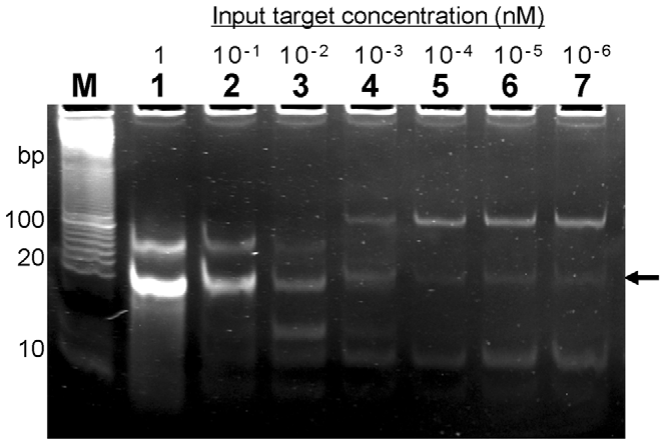
PEAR reactions with different target concentrations. Lane M: Invitrogen Trackit™ 10 bp DNA ladder; lane 2–8: PEAR reaction with 1 to 10^−4^ nM of input oligonucleotides. The lower band (shown by an arrow) represents the 20-bp duplex monomers, and the upper bands represent tandem repeats; Probe (X′R′X′R′X′) concentration is at 100 nM. A final incubation at 75°C for 30 min was conducted to cleave the product.

To determine the sensitivity of PEAR, a series of reactions were conducted with target concentrations ranging from 0.1 pM to 1 nM and probe concentration at 100 nM. As shown in Fig 3, the yield is very high when target concentration was at 0.1 to 1 nM, whereas relatively low when target concentration was less than 10 pM.

### Hpy99I Digestion of the PEAR Product

**Figure 4:**
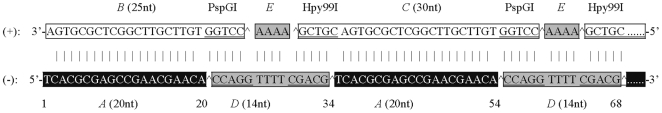
Double digestion of the PEAR product by PspGI and Hpy99I. The sense and the antisense strand are indicated respectively by (+) and (**−**). Recognition sites for PspGI and Hpy99I are underlined and marked. Each position where cleavage is expected to occur is indicated by a caret (“** ^**”). The antisense strands (*A*) are black boxed, the sense strands (*B* and *C*) are boxed, and the by-products (*D* and *E*) are grey boxed. The expected length for each strand is indicated in parenthesis.

Although duplex oligonucleotides showed increased cellular uptake when compared to single-stranded (ss) antisense oligonucleotides [8], and demonstrated improved *in vitro* potency and stability compared to small interfering RNA [9], single-stranded antisense oligonucleotides have been more frequently used in practical applications [10]–[13]. Therefore, it is necessary to separate the antisense oligonucleotides from its complementary strands. To facilitate the separation of the two complementary strands, we introduced a recognition site for restriction enzyme Hpy99I into the probe positioned between the tandem repeats and downstream to the recognition site for PspGI. At the end of PEAR, PspGI cleavage of the product has resulted in a 5-nt overhang in the 5′-end of the sense strand. After PEAR amplification, the products were pooled and further digested with Hpy99I, resulting in another 5-nt overhang in the 3′-end of the sense strand (Fig 4). It is noted that the antisense strand is cleaved into 20-nt monomers, and the sense strand is cleaved into monomers that are 25 or 30 nt in length, which allows convenient separation by chromatographic methods.

### Separation and Analysis of the Antisense Oligonucleotides

**Figure 5:**
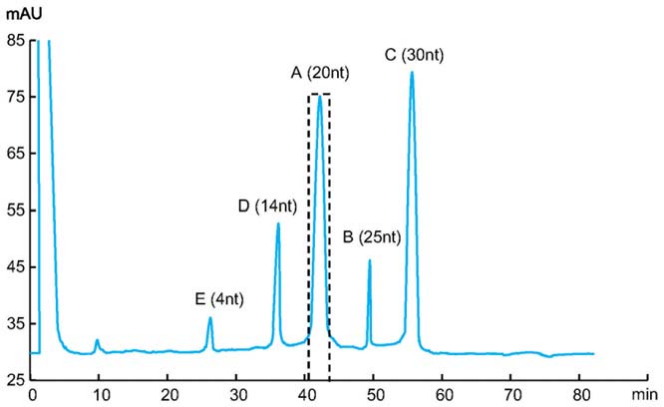
HPLC separation and purification of antisense oligonucleotide. Fractions are indicated by letter A to E as shown in Fig. 4. Fraction A, which contains the antisense strands, was collected in the indicated interval. Sample preparation: 10 µg PEAR product double digested by PspGI and Hpy99I; Column: SOURCE Q PE 4.6/100; Flow rate: 1 ml/min; Buffer A: 10 mM NaOH, pH 12; Buffer B: 10 mM NaOH+2M NaCl, pH 12; Gradient: 20–35% B in 50 column volume.

**Figure 6:**
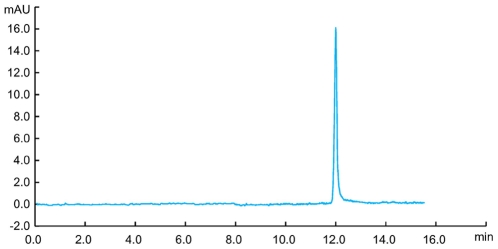
Analytical HPLC analysis of purified antisense oligonucleotide. Sample preparation: 25 µL purified antisense oligonucleotide; Column: DNAPac PA-100 (4/250); Flow rate: 1 ml/min; Buffer A: 10 mM NaClO_4_+1 mM Tris; Buffer B: 300 mM NaClO_4_+1 mM Tris; Gradient: 10–70% B, 7.6 CV.

**Figure 7:**
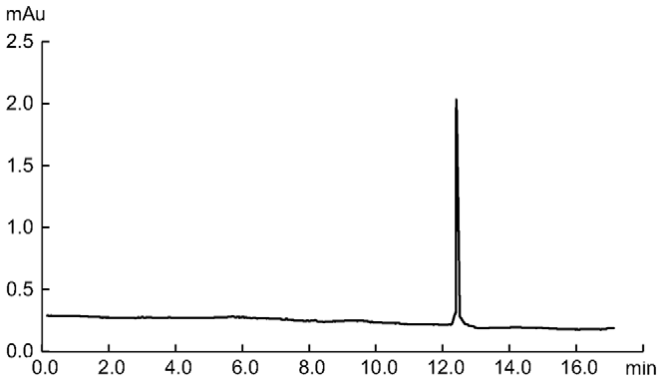
Capillary electrophoresis of HPLC purified antisense oligonucleotide. Capillary: 50 µm×18 cm, filled with polyacrylamide cyclodextrin gel; Buffer: Tris-borate/Urea; Running conditions: 1.5 kV/30 min; Sample application: 1 kV/5 s.

**Figure 8:**
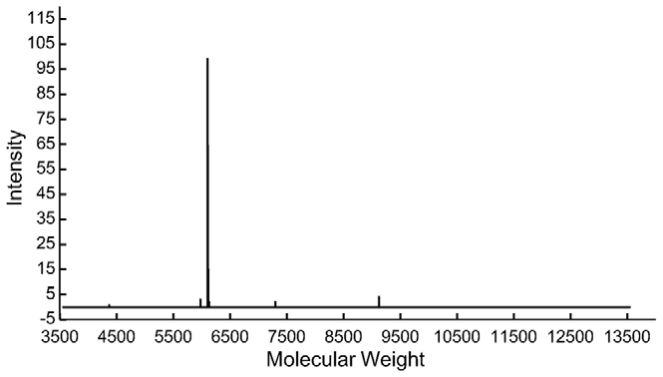
ESI Mass spectrum analysis of HPLC purified antisense oligonucleotide. Calculated molecular weight is 6105.0 Daltons, the measured molecular weight is 6105.5 Daltons.

For separation of the antisense oligonucleotides, we used an anion exchange chromatography using a SOURCE 15Q column [14]. The high pH stability of SOURCE 15Q allows facile separation of the antisense oligonucleotides from its complementary strands under denaturing alkaline conditions at pH 12. The chromatogram shown in Fig 5 represents the purification of 1 nmol of antisense oligonucleotides. The antisense fraction was collected within the window indicated in Fig 5. The 20-nt antisense oligonucleotides were separated from the sense strands, short by-products and remaining tandem repeats. Collected fraction was further analyzed by anion exchange chromatography and capillary electrophoresis. As shown in Fig 6, the purity of the collected fraction analysis by chromatography was >99.0%. Fig 7 shows analysis of purified antisense oligonucleotide by capillary electrophoresis. The purity was confirmed to be better than 99.0%. The purity level of this oligonucleotide product is significantly improved when compared to that of HPLC-purified synthetic oligonucleotides [14]. This is primarily due to the avoiding of highly homologous impurities, especially (n-1) deletions. Further analysis by Electrospray mass spectrometry (ESI-MS) proved that the structure of the purified product is correct (Fig 8).

### Scale-Up and Productivity Evaluation

PEAR reactions were run in 96-well plates, with 95×100 µL PEAR reactions and a no target control (NTC) in each run. To demonstrate the ability to scale up, three successive rounds of PEAR were conducted, at scales of 1, 100 and 500 runs, respectively. The product of round 1 was used diluted 1∶100 as seeds for round 2 directly without any treatment, and that of round 2 for round 3. After cleavage and purification, the quantity of purified antisense oligonucleotide was given by measuring its optical density at a wavelength of 260 nm. The average concentrations, purities, yields and total yields of recoverable (purified) products are estimated and summarized in Table 1. On average, the concentration of purified product is about 10 µM or 70 ng/µl, which is ∼100-fold higher than the input concentrations. The average yield of purified product of a single run is about 0.67 *mg*, which is greater than the typical yield of HPLC-purified product of a 1-µmole scale commercial synthesis. The total yield of round 2 (a 100-fold scale-up of round 1) was increased to ∼67 mg; and that of round 3 (a 5-fold scale-up of round 2), was further increased to ∼336 mg, indicating that this method is well suited for scaling up.

**Table 1:** Average recoverable concentration, purity and yield of the purified antisense oligonucleotide.

Round#^a^	Runs^b^	Average recoverable concentration		Purity^d^	Recoverable Yield (mg)	
		(µM)	(ng/µl )		Average	Total
1	1^c^	10.543	70.966	99.18%	0.674	0.674
2	100	10.579	71.209	99.17%	0.676	67.649
3	500	10.524	70.840	99.17%	0.673	336.490

a The product of round 1 was used as seeds for round 2, and that of round 2 for round 3.

b Each run consisted of 95×100 µl reactions. Target and probe concentration are at 1 nM and 100 nM respectively.

c Round 1 had two duplicate runs. As the first run had been used as seeds, the second run was used for purification and analysis.

d Purity was calculated as peak area % at UV 260 nm.

## Discussion

MiRNAs plays an important role in post-transcriptional gene silencing that is highly conserved among species [1]. Synthetic antisense oligonucleotides to target miRNAs or their target mRNAs have been proved to be powerful tools to specifically and selectively regulate gene expression, and to investigate the roles of dysregulated genes in human diseases [2]. MiR-375, for example, is highly expressed in pancreatic islets and is required for glucose homeostasis. This miRNA is conserved among zebrafish, mice and human [15], [16]. Obese mice exhibit increased miR-375 expression, and genetic deletion of miR-375 resulted in a severe diabetes [17]. Targeted inhibition of miRNA with a modified antisense oligonucleotide reveals the important role for miR-375 in pancreatic islet [18].

Antisense oligonucleotides to target miRNAs or mRNA are expected to be capable of curing a wide variety of human diseases, including cancer, obesity, cardiovascular and metabolic diseases [3], [4] and viral infections [19]. By far, antisense oligonucleotides are all manufactured by solid-support phosphoramidite-coupling chemistry [8]–[14]. Dichloromethane (CH_2_Cl_2_), a halogenated solvent, was used to dissolve the deblocking reagent (trichloroacetic acid, TCA, or dichloroacetic acid, DCA). As oligonucleotide synthesizers are now available for syntheses in large scale, the quantity of chlorinated waste generated becomes quite large [20].

Typically, crude products from solid-support organic synthesis are contaminated with a significant fraction of highly homologous failure sequences that arise from incomplete detritylation, coupling, sulfurization, capping or deprotection [21]. The most significant impurities found in synthetic oligonucleotides are the (n-1) deletions that differ from the full-length product by lacking only one of the desired nucleotides. For oligonucleotides 18–21 nucleotides in length, it is difficult to remove the (n-1) deletions completely from full-length oligonucleotides by HPLC [21].

As a novel and simple method for the amplification of short oligonucleotides, PEAR is capable of solving all of the problems mentioned above. The basic raw materials, nucleotides and enzymes, are all safe and pollution-free. Hence, although a small-scale synthesis of the seeds is still a prerequisite step, the use of hazardous chemicals is reduced to a minimum. Moreover, purification of the PEAR amplified oligonucleotide product is quite routine and easy. The purity level of the product is also improved due to avoiding of (*n*-1) deletions. The error rate of different DNA polymerases has effects on the mutational rate of amplified large DNA fragments. Since the error rates of these thermostable DNA polymerases are very low (typically ranging from 10^−4^ for Taq DNA polymerase to 10^−6^ for high fidelity thermostable DNA polymerases), and the target oligonucleotides amplified in PEAR is very short, the error rate of thermostable DNA polymerases should have minimal effects on the fidelity of the amplified DNA.

For the majority of existing DNA amplification technologies, including polymerase chain reaction (PCR) [22], ligase chain reaction (LCR) [23], rolling circle amplification (RCA) [24], loop-mediated isothermal amplification (LAMP) [25], strand displacement amplification (SDA) [26] and helicase-dependent amplification (HDA) [27], they all need a pair of short synthetic primers to achieve exponential amplification. The yields of the final products are inherently dependent on and limited by the input primer concentrations.

Exponential amplification reaction (EXPAR) is a fast isothermal reaction that can achieve 10^6^-fold amplification of a target oligonucleotide in a short time (less than 10 min) [28]. However the utility of EXPAR is currently limited, as serious nonspecific background amplification was reported [29]. Moreover, obviously in EXPAR DNA synthesis proceeds in only one of the two strands (the target strand), but not in the other (the antisense strand), so the product yield is still limited by the input concentration of the antisense template.

Noteworthy, rolling-circle replication (RCR) that proceeds in a linear fashion using the highly processive phi29 DNA polymerase can copy a circular probe into a DNA strand containing >1,000 tandem repeated complements of a circularized DNA molecule. Circle-to-circle amplification (C2CA) [6], a multi-step RCR-based process for strand-specific amplification of circularized DNA, has been used for the amplification of DNA circles, in which tandem repeated complements of DNA circles are generated by RCR, and converted to monomeric circles of opposite polarity to that of the starting material. Billion-fold amplifications were achieved through successive rounds of ligation, RCR and cleaving. However, a laborious cleavage, ligation and recircularization process are required for each round of RCR amplification. Moreover, a synthetic *ligation template* is required to recircularize and monomerize the tandem repeated complements, so this technique requires the additional production of large amounts of ligation templates. This problem was eliminated by using a hairpin-containing self-templating oligonucleotide that contains a *suicide cassette*, a recognition site for a nicking enzyme, and recircularizing and monomerizing the tandem repeated complements by nicking the suicide cassette [7]. But a laborious nicking, ligation and recircularization process is still needed for each generation of RCR, making it not readily amenable to automation.

In contrast, PEAR is a simple but effective method for the amplification of short oligonucleotides through the unique slipping-and-cleaving mechanism. PEAR uses much less expensive thermocyclers instead of highly expensive DNA synthesizers, so it has the potential to be a useful tool for large-scale production of antisense oligonucleotide drugs. One round of PEAR amplification can result in over 100-fold increment in product yield as evidenced from the data presented here. We have observed more than 1000-fold increment in PEAR reactions (data not show), but we adopted a one hundred-fold amplification for the scale-up reactions in this study, because it provides more reliable and uniform product yield. Three rounds of PEAR starting from micrograms of seeds produced more than three hundred milligrams of products. Thus more rounds of PEAR starting from these hundred-milligram seeds can yield dozen-grams, and then kilograms, of products. Thus, one hundred-fold scale-up is sufficient for large-scale oligonucleotide production.

In this preliminary study, we demonstrated only the production of an unmodified antisense oligonucleotide. Modified antisense oligonucleotides, such as phosphorothioate oligodeoxynucleotides [19], 2′-O-methoxyethyl [10]–[12], 2′-O-methyl [11]–[12] or 2′-fluoro substituted oligonucleotides [11]–[12], and locked nucleic acid [11]–[13], are reported to demonstrate increased stability and potency when compared to its native counterparts. Previously, modified DNA bearing 5(methoxycarbonylmethyl)-2′-deoxyuridin has been prepared in large scale by PCR and post-synthetic derivatization [30]. LNA-modified DNA has been prepared by primer elongation [31], [32], PCR and *in vitro* transcription [33]. The utilization of PEAR technology for the preparation of modified oligonucleotides still needs further study and optimization.

We have tested a number of other thermostable restriction endonucleases such as ApeKI and PhoI that can survive through repeated cycles of high temperature. We found that PspGI is the best choice for PEAR reaction. The enzymatic properties of PspGI have been studied extensively [34], [35]. We are in the process of evaluating a number of different thermostable DNA polymerases such as DyNAzyme II Hot start DNA polymerase and Phusion DNA polymerase that carry the 3′-5′ proof-reading activity and lack the template-independent terminal transferase activity.

## Materials and Methods

### Enzymes and Oligonucleotides

Taq DNA Polymerase, PspGI and Hpy99I, were purchased from New England Biolabs (NEB), Inc. A target oligonucleotide and two antisense probes targeting human microRNA miR-375 were custom synthesized and HPLC-purified by Invitrogen Life technologies. The sequence of the target oligonucleotide is 5′-TGT TCG TTC GGC TCG CGT GA-3′. Both of the two probes contain three tandem repeated complements of the target sequence (X′). The first probe (X′R′X′R′X′) was used for validation of the reaction mechanism, in which the three complements are separated from each other by a recognition site (R′) for PspGI. The second probe (X′R′H′X′R′H′X′) was used in the scale-up reactions, in which two recognition sites (R′ and H′), respectively for PspGI and Hpy99I, are embedded between the repeats. The sequence of the first probe is 5′-TCA CGC GAG CCG AAC GAA CAC CAG GTC ACG CGA GCC GAA CGA ACA CCA GGT CAC GCG AGC CGA ACG AAC A-3′, and that of the second probe is 5′-TCA CGC GAG CCG AAC GAA CAC CAG GTT TTC GAC GTC ACG CGA GCC GAA CGA ACA CCA GGT TTT CGA CGT CAC GCG AGC CGA ACG AAC A-3′. The purity of the target oligonucleotide and probes received was ∼95.0%. They were further purified in our own lab by anion exchange chromatography to achieve >99.0% purity.

### PEAR Reactions

PEAR were carried out in 96-well plates on an Applied Biosystems 9700 Thermal Cycler, each in a 100 µL volume reaction mixture containing 200 µM each dNTP, 15 mM Tris-HCl, 30 mM KCl, 5 mM (NH_4_)_2_SO_4_, 2.5 mM MgCl_2_, 0.02% BSA, 0.08 u/µl Taq DNA polymerase, 0.4 u/µl PspGI restriction enzyme, desired amount of target oligonucleotide and antisense probe. The reactions were initiated at 95°C for 1 min, followed by 30 cycles of denaturing at 94°C for 15 sec, annealing at 55°C for 35 sec, elongation and cleaving at 75°C for 5 min. If it is necessary, PspGI digestion of the product is conducted by a final incubation at 75°C for 30 min. PEAR products were separated by 15% non-denaturing polyacrylamide gel electrophoresis (PAGE), and visualized under an ultraviolet illuminator after SYBR Gold staining (Molecular Probes).

### Cleavage, Separation, Purification and Quantification

The PEAR products were pooled and fully digested by the addition of 1 volume of cleavage mixture containing 1× NEBuffer 4, and 1.0 u/µl of Hpy99I. Cleavage reactions were incubated for 3 hours at 37°C and stopped by heat inactivation at 65°C for 20 minutes. Alternatively, in order to remove BSA, Taq DNA polymerases and excess dNTPs, PEAR products were phenol extracted, ethanol precipitated, washed three times with 75% ethanol, dried and resuspended in ddH_2_O, and double digested by the addition of 1 volume of cleavage mixture containing 1× NEBuffer 4, and 1.0 u/µl each of Hpy99I and PspGI. Cleavage reactions were incubated for 3 hours at 37°C and 30 minutes at 75°C. Separation and purification of the antisense oligonucleotide were performed by anion exchange chromatography using ÄKTA explorer™ 10 system (GE Healthcare) as described [14]. SOURCE Q PE 4.6/100 prepacked analytical columns filled with polymer-based, 15 µm monosized beads were purchased from GE Healthcare. The column effluent was fractionated and collected using a Frac-950 Fraction Collector (GE Healthcare). The volume of collected fraction was 0.5 column volume (CV). The chromatographic conditions are described in the legend to Fig 5. A HiPrep™ 26/10 column (GE Healthcare) was employed for desalting the purified oligonucleotide using MilliQ™ ultrapure water (Millipore Corporation, Billerica, USA) as eluent. The mass estimates of the purified antisense oligonucleotide were given by 260 nm absorbance measurements of diluted samples. The data were analyzed in Microsoft Excel.

### Purity Analysis

The purity of purified antisense oligonucleotide was analyzed by anion exchange chromatography as described [14]. The anion exchange HPLC analysis was conducted on Hewlett-Packard Model 1100 liquid chromatography (Palo Alto, CA, USA) fitted with a non-porous medium DNAPac PA-100 4/250 mm analytical column (Dionex Corporation, Sunnyvale, CA, USA), thermostatted to 25C and operated at a flow rate of 480 cm/h. The chromatographic conditions are described in the legend to Fig 6.

Capillary electrophoresis was performed using a 50-µm I.D.×20-cm (effective length 18 cm) capillary. The capillary was treated with PlusOne™ Bind-Silane (GE Healthcare) and filled with polyacrylamide cyclodextrin gel. Buffer was consisted of 0.1 M Tris-0.25 M boric acid, pH 8.5, containing 7 M urea. Injection was made at 1 kV for 5 sec. Analysis was performed at 1.5 kV for 30 min.

### Electrospray Mass Spectrometry (ESI-MS) Analysis

Mass spectrometry analyses were performed on a VG Platform II mass spectrometer (Fisons Instrument) as described in [36]. The N_2_ nebulizing gas was maintained at 100 p.s.i., with a 5.0 l/min flow as drying gas. The HPLC-purified oligonucleotide product was brought up in a 90∶9∶1 methanol∶H_2_O∶NH_4_OH solution at a concentration of 5 pmol/ml. Samples were infused utilizing a Harvard Instruments Syringe Infusion Pump 22 at a flow rate of 10 ml/min. Tests were performed in the negative ionization mode and the ESI source temperature was kept at 120C. Fifteen continuum scans were averaged in the ‘MCA’ mode over the mass to charge range (m/z) 300–1700.
